# Slaving and release in co-infection control

**DOI:** 10.1186/1756-3305-6-157

**Published:** 2013-05-31

**Authors:** Laith Yakob, Gail M Williams, Darren J Gray, Kate Halton, Juan Antonio Solon, Archie CA Clements

**Affiliations:** 1 Infectious Disease Epidemiology Unit, School of Population Health, University of Queensland, Herston, Brisbane, Qld 4006, Australia; 2 Molecular Parasitology Laboratory, Queensland Institute of Medial Research, Herston, Brisbane, Qld 4029, Australia; 3 Institute of Health and Biomedical Innovation, Queensland University of Technology, Kelvingrove, Qld 4059, Australia; 4 College of Public Health, University of the Philippines Manila, 625 Pedro Gil Street, Ermita, Manila, Philippines

**Keywords:** Nematode, Mathematical model, Infectious disease, Epidemiology, Next generation matrix, Drug resistance

## Abstract

**Background:**

Animal and human infection with multiple parasite species is the norm rather than the exception, and empirical studies and animal models have provided evidence for a diverse range of interactions among parasites. We demonstrate how an optimal control strategy should be tailored to the pathogen community and tempered by species-level knowledge of drug sensitivity with use of a simple epidemiological model of gastro-intestinal nematodes.

**Methods:**

We construct a fully mechanistic model of macroparasite co-infection and use it to explore a range of control scenarios involving chemotherapy as well as improvements to sanitation.

**Results:**

Scenarios are presented whereby control not only releases a more resistant parasite from antagonistic interactions, but risks increasing co-infection rates, exacerbating the burden of disease. In contrast, synergisms between species result in their becoming epidemiologically slaved within hosts, presenting a novel opportunity for controlling drug resistant parasites by targeting co-circulating species.

**Conclusions:**

Understanding the effects on control of multi-parasite species interactions, and vice versa, is of increasing urgency in the advent of integrated mass intervention programmes.

## Background

The epidemiological and economic impact of multi-species co-infection is largely unknown and seldom considered in strategising control. However, many pathogens are typically found to co-circulate because hosts are a rare and patchy resource, and because of shared geographical distribution of parasites, and similar transmission routes [1]. The one-host one-pathogen paradigm that constitutes the backbone of infectious disease understanding requires expansion to account for this ubiquitous epidemiological setting. A framework is needed for optimising control when an infectious agent constitutes one of many interacting pathogens. Using gastro-intestinal nematode parasites as an illustrative example, we simulate different control strategies and their effects on infection prevalence when there are multiple, interacting parasites.

Gastro-intestinal nematodes are direct lifecycle parasites of veterinary and human health significance. Economically important nematode parasites of livestock include *Haemonchus* spp., *Teladorsagia* spp. and *Nematodirus* spp., which infect ruminants, *Ascaris suum*, a pig parasite, and *Strongylus vulgaris* and *Strongyloides westeri* in horses, among others. Human worm burden is mainly associated with *Ascaris lumbricoides* (large roundworm), *Trichuris trichiura* (whipworm) and the hookworms *Ancylostoma duodenale* and *Necator americanus*. These soil-transmitted helminths (STHs) infect over 1 billion people, incurring 40 million disability-adjusted life years, mostly in sub-Saharan Africa, East Asia, China, India and South America [2-4]. Gastro-intestinal nematode parasite transmission is through contact with contaminated soil or consumption of contaminated food or water, and co-infection with multiple species is widely-documented in humans and animals [5]. Co-infections have important consequences to parasite ecology [6] and associated host morbidity [1,7], and they warrant careful consideration when strategising control.

Control of gastro-intestinal nematodes is largely dependent on drugs of three main chemical classes: benzimidazoles, imidazothiazoles and macrocyclic lactones. Given the prevalence of co-infection and common approach to control for these parasites, there is increasing momentum in public health towards a model of integration, whereby multiple species are targeted simultaneously [8-13]. However, there are two key issues related to drug dependence that hold implications for this approach. First, chemotherapeutic efficacy is highly species specific. For example, cure rates of STHs following treatment with Albendazole are 88% for *A*. *lumbricoides*, 78% for the hookworms and only 28% for *T*. *trichiura*[14]. Furthermore, drug and multidrug resistance has become rife in livestock infections [15,16] and threatens the success of future mass drug application programmes in humans [17]. Second, the implications for control when host populations are co-infected with multiple, interacting parasite species are largely unknown. We developed a simple epidemiological model of co-infection to explore both these issues.

Given difficulties in obtaining empirical evidence, mathematical models have been developed to fill in data gaps and inform deworming programme strategies [18,19]. Most studies have followed in the footsteps of Anderson and May (1985) by analysing the population biology of a single helminth species [20]. Within-species interactions can be direct (e.g., via resource competition) or indirect (e.g., via immunomodulation) and generally are assumed to act upon two distinct life stages of the parasite: establishment of new infection or adult parasite fecundity [21]. The crux of our investigation, however, lies with the combinations of interactions that can arise from multi-species infections. By investigating the diverse range of interspecific interactions documented in laboratory animals [22-25] and humans [26,27], we discover epidemiological settings that can strongly enhance, attenuate, or even reverse, the intended impact of antihelminthic control programmes. In the advent of integrated parasite control we highlight the importance of developing a more holistic epidemiological framework for understanding the effects of perturbing parasite assemblages.

## Methods

### Rationale for a microparasite approach to modelling macroparasite control

Since Anderson and May (1978), there has been a divergence in the way by which ‘microparasites’ (e.g., viruses, bacteria, protozoa) and ‘macroparasites’ (e.g., nematodes, trematodes) have been modelled mathematically [28]. Typically, microparasite infection transmission is modelled using derivations of the classic SIR (Susceptible Infected Recovered) framework in which rates of change in proportions of the population between the epidemiological compartments are tracked using ordinary differential equations. This provides a constantly updated record of the prevalence of infection. Tracking prevalence of infection has been deemed insufficient for understanding macroparasite epidemiology because these types of parasites are highly aggregated among hosts, with hosts harbouring higher worm burdens (intensity) suffering more severe symptoms. Consequently, studies of macroparasite disease transmission, including co-infection models, have used a negative binomial forcing function to describe the aggregation of parasites within the host population [29-33].

While it would have been convenient to follow these examples, it would not have been appropriate for the intended purposes of our analysis. Key to our study is the interplay between parasite interactions and control. Although the negative binomial parasite aggregation method has proven very useful in describing endemic parasite distribution among hosts in the absence of control, it consists of a post hoc method of forcing the model output to better resemble the endemic equilibrium data. This method is not mechanistic - it does not attempt to describe the mechanism by which parasites become aggregated within host populations. Indeed, the precise mechanisms by which parasites aggregate within their hosts are unknown and likely to vary with species. Of paramount importance is the fact that the different mechanisms by which aggregation comes about will affect, and be affected by, control differently. Descriptive, phenomenological approaches (such as the negative binomial aggregation method) therefore become inappropriate for simulating non-equilibrial conditions, such as control scenarios.

One alternative modelling approach is to use individual-based simulations, such as those described by Bottomley *et al*. (2005) and Fenton *et al*. (2010) [34,35]. In the model of Bottomley *et al*. (2005), exposure to the parasite(s) is indistinguishable from host susceptibility to infection. The authors’ justification for this conflation was that, for their purposes, it did not matter whether a host remained uninfected because they were not exposed to a parasite or because the host was not susceptible to infection. While it has been useful in exploring interspecific effects of parasites at endemic equilibrium, this framework cannot be used for the simulation of control scenarios because the dynamics of free-living parasites and hosts’ infection status/susceptibility require explicit decoupling.

Fenton *et al*. (2010) also adopted an individual based modelling approach. These authors used a fixed lifelong probability of being infected with a particular parasite (with the probability assigned from a negative binomial distribution), in order to explore interspecific interactions during co-infection. This assumption was necessary because “allowing hosts to vary in their infection rates throughout their lifetime could introduce considerable noise to the data, greatly hampering the ability to detect any clear signal of interspecific interaction”. Unfortunately, this methodology cannot be applied to situations in which infection rates are anticipated to vary temporally, such as would be expected during, and following, control programmes. Therefore, to date, there is no modelling framework that can simulate macroparasite co-infection transmission dynamics in order to assess disease mitigation strategies. This was the motivation for our study.

### The mathematical model of parasite co-infection

**Figure 1:**
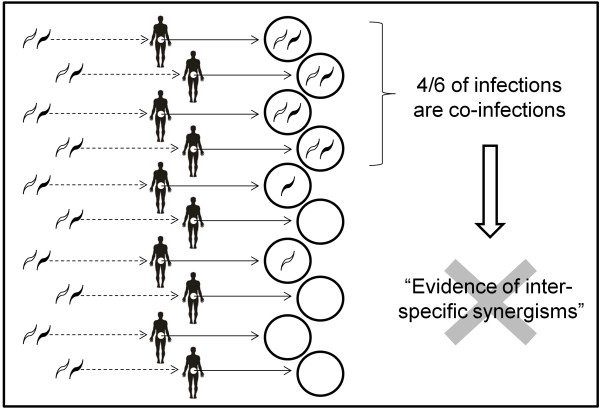
**Observations of co**-**infection prevalence can misrepresent between**-**parasite species interactions.** In the above example, exposure to antagonistic parasites has resulted in no infection, or mono-specific infection, in most hosts. 5/10 people are infected with Parasite 1 (white) and 5/10 people are infected with Parasite 2 (black). More hosts are co-infected (4/10) than might be expected from random chance (5/10 x 5/10 = 2.5/10). This would typically lead to the incorrect assertion that the parasites exhibit synergistic interactions. Only by knowing both the patterns of infection status and exposure to contaminated environment can inferences be made of within-host between-parasite species interactions.

Two co-circulating gastro-intestinal nematode species were simulated using a simple adaptation of classic epidemiological model design whereby the host population was compartmentalised into Susceptible (S), Infected with nematode species 1 (I_1_) or 2 (I_2_) or both (I_12_). Infected hosts contaminate (β) the environment, and the subsequent free-living stages then either infect secondary hosts (λ) or perish (ν). The proportion of the total environment (E) that is contaminated with either (E_1_ or E_2_), or both (E_12_), nematode species was modelled explicitly. Traditionally, contamination of the environment is not modelled explicitly and, for mathematical convenience, exposure of hosts to free-living stages is assumed to be directly proportional to the adult worm population harboured by infected hosts [20]. We explicitly model the environment for three main reasons. First, it incorporates a more realistic time delay between the shedding of eggs and exposure of secondary hosts to infective stages. Second, it allows us to track separately the patterns of exposure and the subsequent infection status of the hosts (the importance of measuring both phenomena is exemplified in Figure 1). Third, explicitly modelling the environment bridges the gap between previous studies and future attempts to simulate more realistic scenarios that include spatial heterogeneities.

Infected individuals can return to the Susceptible class through recovery from infection (γ) under the assumption of no lasting immunity. Table 1 shows the symbology and definitions of the model parameters. Because many nematode infections can be long-lasting, host demographics were incorporated and a stable population was assumed (births = deaths). The set of ordinary differential equations used to describe two nematode species co-circulating within a host population and the environment is as follows:

(1.1) $$\begin{array}{l} dI_{1}/\mathrm{dt}=\lambda_{1}\left(E_{1}+E_{12}\right)S+\gamma_{2|12}I_{12}\\ \;–\left[\gamma_{1}+\alpha_{1}\lambda_{2}\left(E_{2}+E_{12}\right)+\mu +\mu_{1}\right]I_{1} \end{array}$$

(1.2) $$\begin{array}{l} dI_{2}/\mathrm{dt}=\lambda_{2}\left(E_{2}+E_{12}\right)S+\gamma_{1|12}I_{12}\\ \;–\left[\gamma_{2}+\alpha_{2}\lambda_{1}\left(E_{1}+E_{12}\right)+\mu +\mu_{2}\right]I_{2} \end{array}$$

(1.3) $$\begin{array}{l} dI_{12}/\mathrm{dt}=\alpha_{1}\lambda_{2}\left(E_{2}+E_{12}\right)\;I_{1}+\alpha_{2}\lambda_{1}\left(E_{1}+E_{12}\right)I_{2}\\ \;–\left(\gamma_{1|12}+\gamma_{2|12}+\gamma_{12}+\mu +\mu_{12}\right)\;I_{12} \end{array}$$

(1.4) $$\begin{array}{l} dE_{1}/\mathrm{dt}=\left(\beta_{1}I_{1}+\beta_{1|12}I_{12}\right)E\\ \;+\nu_{2}E_{12}–\left(\nu_{1}+\beta_{2}I_{2}+\beta_{2|12}I_{12}\right)E_{1} \end{array}$$

(1.5) $$\begin{array}{l} dE_{2}/\mathrm{dt}=\left(\beta_{2}I_{2}+\beta_{2|12}I_{12}\right)E\\ \;+\nu_{1}E_{12}–\left(\nu_{2}+\beta_{1}I_{1}+\beta_{1|12}I_{12}\right)E_{2} \end{array}$$

(1.6) $$\begin{array}{ll} dE_{12}/\mathrm{dt} & =\left(\beta_{2}I_{2}+\beta_{2|12}I_{12}\right)E_{1}\\ & \;+\left(\beta_{1}I_{1}+\beta_{1|12}I_{12}\right)E_{2}–\left(\nu_{1}+\nu_{2}\right)E_{12} \end{array}$$

**Table 1:** The parameters used in the model along with their definitions and simulated values

**Parameter**	**Definition (all rates are daily)**	**Simulated values**
**P1, P2**	Parasite species 1 and 2	n/a
**S**	Susceptible host proportion	Dynamic
**I**_**1**_**, I**_**2**_**, I**_**12**_	Host proportions infected with P1, P2 and both	Dynamic
**E**_**1**_**, E**_**2**_**, E**_**12**_	Environment contaminated with P1, P2 and both	Dynamic
**λ**_**1**_**, λ**_**2**_	Rate at which new infections are established	0.1216328
**β**_**1**_**, β**_**2**_	Rate at which hosts contaminate the environment	1.216328x10^-2^, x10^-3^
**β**_**1|12**_**, β**_**2|12**_	Rate at which co-infected hosts contaminate the env.	0.1-10 x β_1_, x β_2_
**α**_**1**_**, α**_**2**_	Relative susceptibility to heterologous parasite infection	0.1-10
**μ**	Natural mortality rate of hosts (~50 yr life expectancy, parasite-induced host mortality can be modelled by μ_i_ > 0)	5.479452x10^-5^
**ν**_**1**_**, ν**_**2**_	Mortality rates of the free-living P1 and P2 stages	0.1
**γ**_**1**_**, γ**_**2**_	Host recovery rate from P1 and P2 infection (~2 yr)	1.369863x10^-3^
**γ**_**1|12**_**, γ**_**2|12**_	Recovery rate from P1 and P2 infection when co-infected	1.369863x10^-3^

Parameterisation was consistent with major human infections with gastro-intestinal nematodes and longevity of free-living stages mostly reflects hookworm biology [36,38,39]. For comparison purposes, transmission rates in the absence of between-parasite interactions were set to ensure a basic reproduction number of 1 (for P2) and 10 (for P1), but were allowed to vary across a wide range of interspecific effects. Chemotherapy was simulated by increasing the host recovery rate(s); sanitation was simulated by reducing the rate at which hosts contaminate the environment.

Where, S = 1 – (I_1_ + I_2_ + I_12_) under the assumption that hosts are born infection-free, and E = 1 – (E_1_ + E_2_ + E_12_). Our analysis explored the effect of synergistic and antagonistic interspecific interactions by adjusting the susceptibility to heterologous infection (respectively, α_1_ or α_2_ >1; α_1_ or α_2_ <1) and by adjusting the fecundity of heterologous parasites during co-infection (respectively, β_1|12_ or β_2|12_ >1; β_1|12_ or β_2|12_ <1).

A key characteristic of gastro-intestinal nematode epidemiology is the overdispersed distribution of parasites within a population: a minority of individual hosts harbour the majority of adult worms [36,37]. Reasons for this clumping are incompletely understood but are normally attributed to genetic predisposition, between-individual immunological differences and/or heterogeneities in behavioural risk factors [40]. Experimental work carried out with numerous parasite species has demonstrated strongly negative density-dependent effects, whereby worm survival and/or fecundity is compromised within hosts suffering higher burdens [41-43]. Other mathematical studies have described in great detail these single-species intra-specific effects on parasite population biology [20,21,44]. Higher per capita proliferation experienced at lower worm burdens has the inevitable effect of reducing the extent of heterogeneity in individual-host level parasite transmission potential that would otherwise be expected [45,46]. This generalisation facilitates our analysis by allowing us to focus on the prevalence (and not the heterogeneous intensities) of multiple parasite species that are co-circulating at the host-population level. Moreover, it provides a framework that is more analytically tractable and, therefore, more adaptable to a wider range of specific scenarios. An in-depth, individual-based, stochastic mathematical exploration of intensity-prevalence relationships in multi-helminth infections has been described elsewhere [34]. Further, in the context of an elimination programme, it will be the trickling effect of drug-attenuated low-intensity infections that perpetuates these parasites, as high-intensity infections will become vanishingly rare and substantially less influential on the population-level epidemiology.

Starting with the simplest case in which there is only one parasite species (Parasite ‘*i*’), the basic reproduction number is the product of the rate of environmental contamination and rate of host infection (accounting for host recovery as well as host and parasite mortality):

(2) $$R_{i}=\left(\beta_{i}\lambda_{i}\right)/\left[\nu_{i}\left(\gamma_{i}+\mu +\mu_{i}\right)\right]$$

Because we will be using next generation matrix methods for outbreak threshold calculations in the more complex stages of this analysis (Supporting Material), the above expression actually corresponds to the squared dominant eigenvalue of the next generation matrix. In order to calculate the invasion threshold of P2 following its introduction into a host population that already harbours P1 at endemic equilibrium, the rates of change for E_1_ and I_1_ are set to equal zero and the resulting simultaneous equations are then solved, leading to:

(3.1) $$­{Î}_{1}=\left[\beta_{1}\lambda_{1}–\nu_{1}\left(\mu +\mu_{1}+\gamma_{1}\right)\right]\;/\left[\beta_{1}\left(\lambda_{1}+\mu +\mu_{1}+\gamma_{1}\right)\right]$$

(3.2) $${Î}_{1}=1-\;\left[\nu_{1}\left(\lambda_{1}+\mu +\mu_{1}+\gamma_{1}\right)\right]\;/\left[\lambda_{1}\left(\nu_{1}+\beta_{1}\right)\right]$$

The hats over Î and Ê indicate that they are at their endemic equilibrium level. These can then be incorporated into the outbreak threshold calculation:

(4) $$\begin{array}{ll} R_{2|1} & =\left[\left(\beta_{2}\lambda_{2}\left(1-{Î}_{1}\right)\right)\right)/\left(\nu_{2}\left(\gamma_{2}+\mu +\mu_{2}\right)\right)\\ & +\left(\beta_{2|12}\lambda_{2}{Î}_{1}\left(1-\gamma_{1|12}\right)\right)/\left(\nu_{2}\left(\gamma_{12}+\gamma_{2|12}+\mu +\mu_{2}\right)\right)\\ & +\left(\beta_{2}\lambda_{2}{Î}_{1}\gamma_{1|12}\right)/\left(\left(\nu_{2}\left(\gamma_{2}+\mu +\mu_{2}\right)\right)\right]\\ & x\;\left[\left(1-{Î}_{1­}\mathrm{­­­}\right)+\alpha_{1}{Î}_{1}\right] \end{array}$$

In words, the introduced P2 either manifested in a susceptible host (1st term within square brackets), in a host already infected with P1 (2nd term) or a host that has since cleared P1 following P2 establishment (3rd term). This introduced case can then go on to infect a susceptible host (1st term in second set of square brackets) or a host already carrying P1 (2nd term). Initially, only the uni-directional effect of P1 on P2 was explored (α_2_ = β_1|12_ = 1); later scenarios allowed for bi-directionality in parasite interactions (Supporting Material).

The design of the model means that it is also applicable to the exploration of within-parasite species i.e. different strains of same species, instead of different species, interactions. Asymmetric competitive interactions between strains can be used to explore evolutionary selectivity with differences in susceptibility to chemotherapy representing the development of drug resistance. In this circumstance, calculations of the successful spread of a secondary parasite strain into a host population already endemic for a primary strain (R_2|1_) demonstrate the parasitological life history trade-offs that need to be negotiated in order for drug resistance to spread.

## Results

Synergisms and antagonisms in animal and human co-infection are typically inferred through (co-)infection prevalence data. Figure 1 illustrates how this can misrepresent the true nature of between-pathogen interactions, which can only be achieved with knowledge of exposure levels, as well as host infection status. We demonstrate the profound public health implications posed by this popular misconception with a simple mathematical model that simulates the attempted intervention of two co-circulating parasite species.

### Unidirectional effects of Parasite species 1 on Parasite species 2

**Figure 2:**
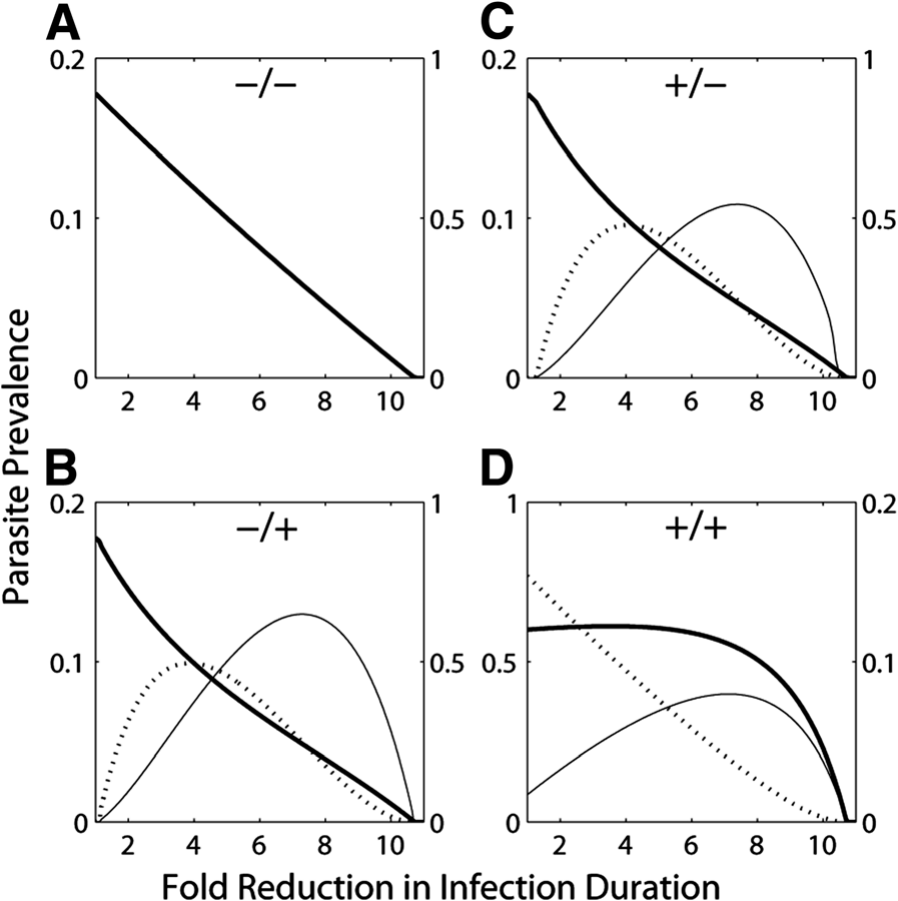
**Parasite prevalence following chemotherapeutic control of P1.** P2 and co-infection are the solid and broken lines on the left y-axis, P1 is the thick black line on the right y-axis. The between-parasite species interactions for these four scenarios are: **A**) P1 reduces both host susceptibility to, and fecundity of, P2 (−/−); **B**) P1 reduces host susceptibility to, but increases fecundity of, P2 (−/+); **C**) P1 increases host susceptibility to, but reduces fecundity of, P2 (+/−); **D**) P1 increases host susceptibility to, and increases fecundity of, P2 (+/+).

First, we consider a co-infection transmission model that only accounts for one direction of interactions (the effects of parasite species 1 ‘P1’ on parasite species 2 ‘P2’). Through host immunological modulation and/or direct resource competition, an extant parasite can enhance or suppress both the host susceptibility to, and the fecundity of, heterologous parasites. Figure 2 shows the four combinations of antagonism/synergism for P2 establishment/fecundity resulting from interaction with P1 (the effect on outbreak thresholds is shown in Additional file 1: Figure S1). The prevalence of P1, P2 and co-infections are plotted for a range of P1-specific control levels. This simulates a species-specific infection treatment or, alternatively, the administering of a drug to which P2 is resistant. When P1 facilitates the establishment of P2 infection as well as its subsequent fecundity, the pathogens become epidemiologically slaved whereby the disproportionate majority of infections are tethered in co-infections (Figure 2D). ‘Allee effects’ are then experienced by P2 whereby its prevalence diminishes with increasing levels of P1 control. In other words, inadvertent suppression of a secondary pathogen occurs by the administration of a drug that would be assumed to have no effect in a one-host one-pathogen mindset.

The effect of intervention on (co-)infection prevalence is very different when mixed synergistic/antagonistic pressures are exerted by P1 (Figure 2B,C). Here, moderate levels of P1 control exacerbate the prevalence of P2 as well as co-infections. This is of major concern given the enhanced morbidity often associated with co-infection [7]. There is a trade-off between P2 being released from suppression by P1 at one stage of its lifecycle and its transmission being enhanced at a different stage. Therefore, when elimination is unachievable, infectious disease suppression is not necessarily the next-best option; the pros of suppressing the more prevalent parasite must be weighed against the cons of potentially exacerbating co-infections and associated co-morbidities.

### Bidirectional effects between Parasite species 1 and Parasite species 2

**Figure 3:**
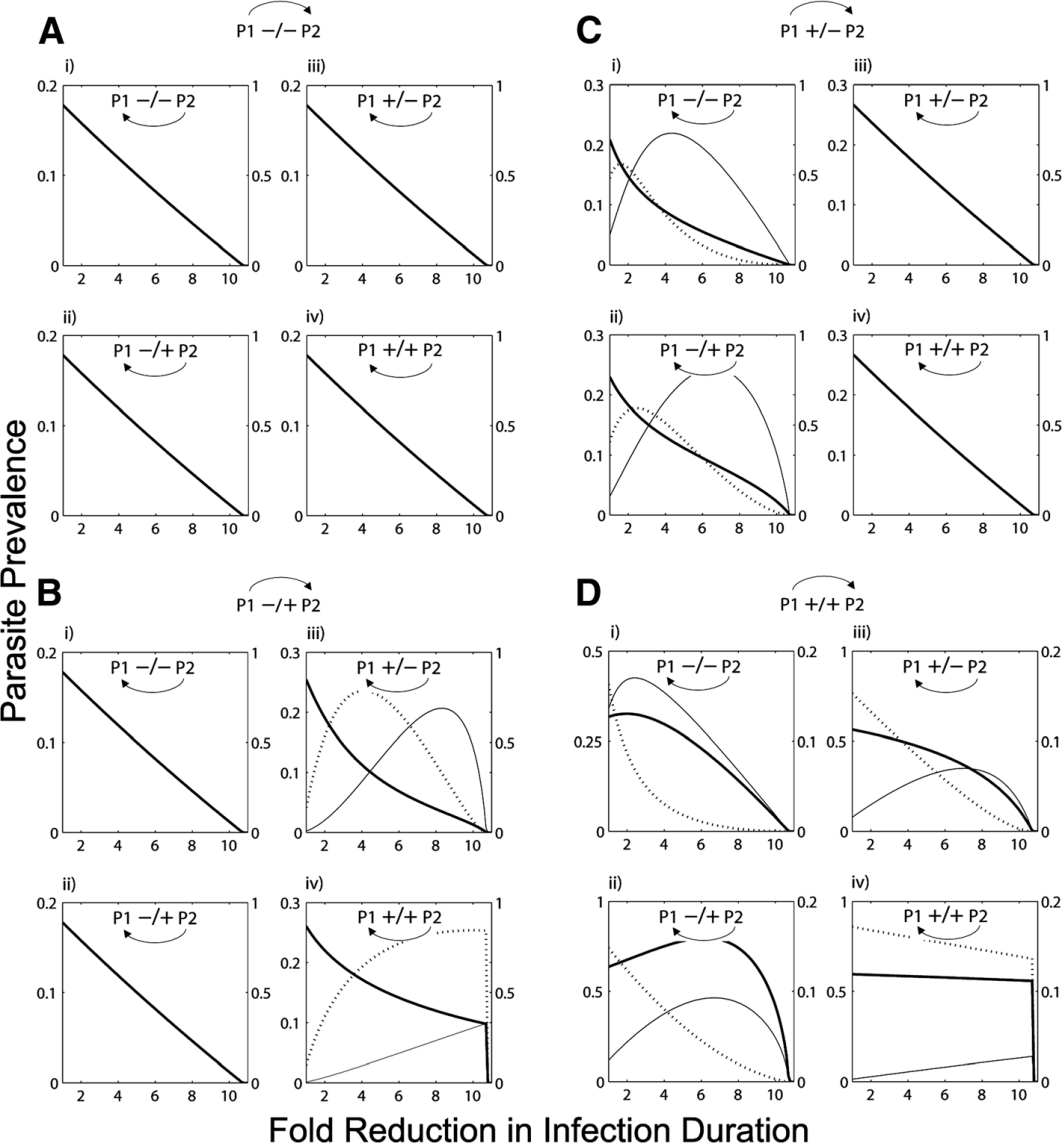
**Parasite prevalence under control of Parasite 1 and allowing for bi**-**directionality in their effect.** P2 and co-infection are the solid and broken lines on the left y-axis, P1 is the thick black line on the right y-axis; the x-axis is the fold reduction in Parasite 1 infection duration. The effect of Parasite 1 on Parasite 2 is depicted by the four larger quadrants: **A**) P1 reduces both host susceptibility to, and fecundity of, P2 (−/−); **B**) P1 reduces host susceptibility to, but increases fecundity of, P2 (−/+); **C**) P1 increases host susceptibility to, but reduces fecundity of, P2 (+/−); **D**) P1 increases host susceptibility to, and increases fecundity of, P2 (+/+). The equivalent effect of Parasite 2 on Parasite 1 is represented in the smaller sub-plots (respectively i, ii, iii and iv).

**Figure 4:**
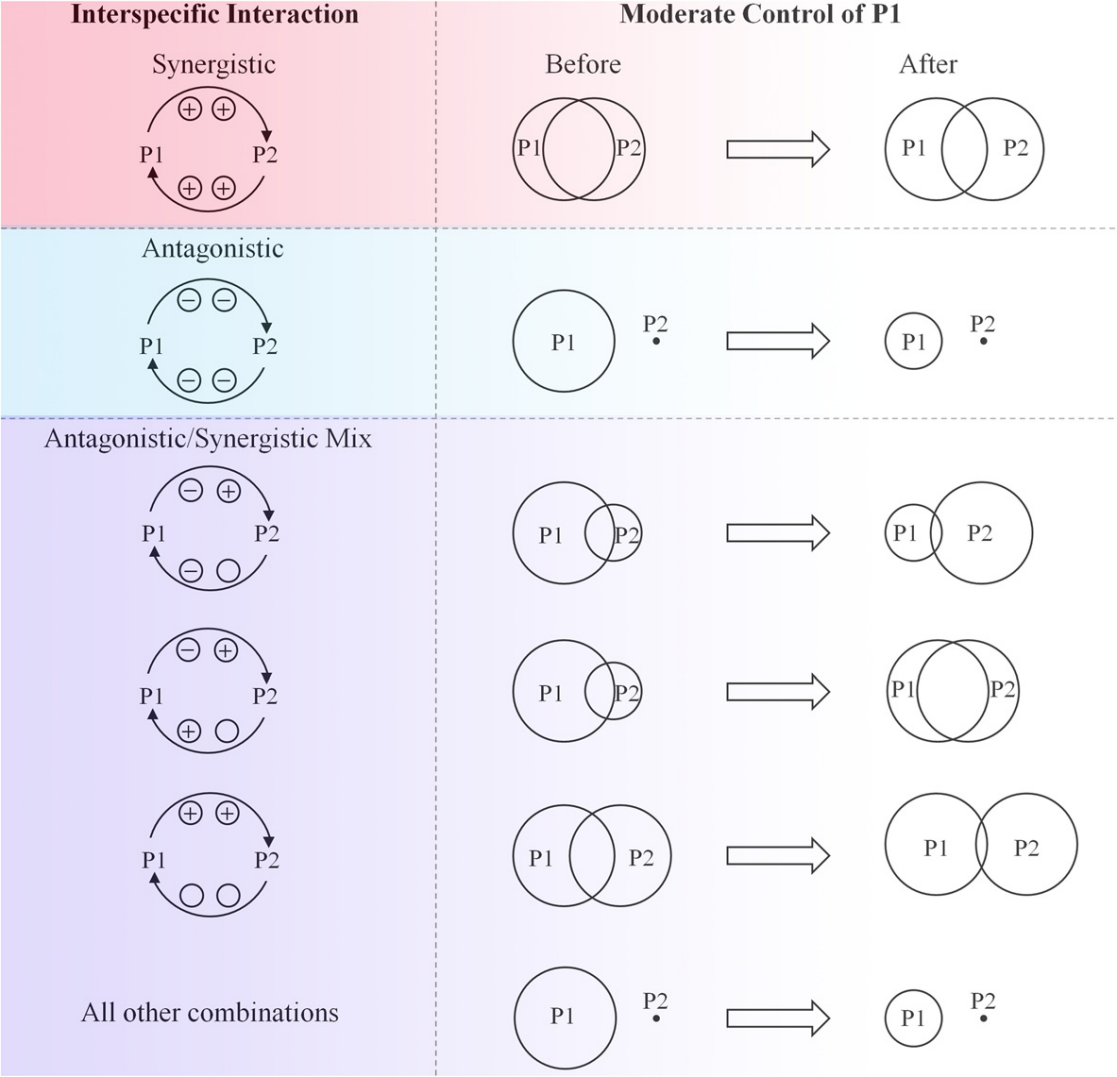
**The qualitative effects of moderate P1 control are dependent on interspecific interactions between P1 and P2.** “Moderate control” is 50% of the level of chemotherapeutic control required for elimination. Interactions can be synergistic (+) or antagonistic (˗) and can occur at different stages of the transmission cycle (the left interaction symbol denotes host susceptibility to heterologous infection, and, the right symbol is fecundity of heterologous adult parasites following infection establishment). Where the polarity of these interactions does not affect qualitative behaviour, the interaction is left blank. The prevalence of P1 and P2 before and after moderate P1 control is represented by the circle size, and the prevalence of co-infection by the amount of intersection. Where P2 fails to persist, it is represented by a point. To exemplify the link between this conceptual figure and the corresponding simulation results, the purely synergistic scenario (top interaction) corresponds to half way across the x-axis of Figure 3Div, and the purely antagonistic scenario (second from top interaction) corresponds to half way across the x-axis of Figure 3Ai.

Until now, we have only considered uni-directional pathogen interactions (the effect of P1 on P2). However, incorporating bi-directionality is important because, typically, nematode co-infections are neither uni-directional, mutually synergistic nor mutually antagonistic, but rather, a more complex blend [47-49]. As a result of this additional flexibility, the number of qualitatively distinct combinations increases, resulting in a richer diversity in epidemiological outcomes. Figure 3 shows the prevalence of these pathogens following treatment of the host population for P1 infection. When the parasites are mutually beneficial (Figure 3D iv), drug control of P1 is attenuated, necessitating aggressive levels of P1 control before any drop in overall prevalence is actually achieved. In accordance with the simpler, uni-directional simulations, there are non-monotonic effects between control effort and pathogen transmission for mixed synergistic/antagonistic combinations (e.g., Figure 3B iii), and moderate levels of control risk increasing the overall prevalence of co-infections. The qualitatively different responses of co-circulating parasite epidemiology following moderate P1 control are summarised in Figure 4. In general, three different adverse outcomes can result from attempting P1 control in a co-infection system: 1) The presence of co-circulating P2 can buffer the effect of chemotherapeutics, providing an indirect resistance of P1 to control (when the parasites are mutually synergistic e.g., top scenario of Figure 4); 2) Controlling P1 can result in the competitive release of P2 whose transmission would otherwise be suppressed by P1 (when P1 reduces host susceptibility to heterologous infection but enhances heterologous parasite fecundity e.g., top scenario of synergistic/antagonistic mix of Figure 4); and, 3) Co-infection exacerbation can occur (when the previous situation also involves a positive effect of P2 infection on the host susceptibility to P1). This last combination is the worst case scenario as it yields all three adverse effects.

Chemotherapeutic control using drugs that are equivalently effective on both pathogens, and reducing environmental contamination with pre-adult parasite stages by improving sanitation, yield very similar results to each other (Additional file 1: Figure S2 & S3). The range of epidemiological patterns is constrained when controls have symmetrical effects on each of the pathogens. In general, presence of a secondary parasite only affects control of the dominant parasite when it enhances its transmission. Drugs and sanitation then more effectively control P2 (the pathogen with a lower intrinsic rate of transmission) and co-infections, while suppression of P1 is not achieved until the heterologous pathogen is eliminated.

## Discussion

The results we present encompass a broad continuum of interspecific interactions. Determining the extent to which co-infecting parasite species interact in nature is not trivial. Using log-linear regression, Howard *et al*. (2001) analysed metadata collected on 215000 individuals in Africa and Asia [50]. Although most significant associations between the four main STH species were positive, significant negative associations were also found for each of the different combinations of co-infection. Poulin (2005) and Fenton *et al*. (2010) recently described the inherent difficulties involved in tweezing apart macroparasite interactions from ecological data [35,51]. These authors suggest that current methods that are routinely implemented for detecting interactions are unreliable and likely underestimate the extent to which they occur in reality. Moreover, ecological data of association cannot distinguish between the different mechanisms of interaction occurring at different stages of the transmission cycle – a feature that we and a previous, single species modelling study demonstrate to be critical in determining the impact of control [21].

Much of our understanding, therefore, comes from experimental animal models. Cox (2001) has compiled a comprehensive list of different types of mixed-species parasite interactions mediated by the immune systems of animal models [5]. Examples include *Trichuris muris* and *Heligmosomoides polygyrus* in which the trichurid benefits during mouse co-infection, and *Schistosoma mansoni* (a trematode) and *Strongyloides venezuelensis* (a nematode) resulting in a detrimental effect for the nematode [52,53]. However, it is difficult to know precisely how representative these animal models are of real-world epidemiology when qualitatively inconsistent findings are obtained from highly related mammalian host species [54,55].

Efforts to ameliorate morbidity and mortality from infectious disease require a shift in the way in which pathogenic transmission is conceptualised. The one-host one-parasite epidemiological paradigm does not apply in a world where infections are largely multi-host and concomitant [1,56]. The mathematical extensions to epidemiological models used in this study are very simplistic; it was our intention to demonstrate transparently the unintuitive, and sometimes counter-intuitive, repercussions of perturbing a pathogen when it constitutes merely a single component of a more complex community. The complex spectrum of qualitative behaviours presented here will only become increasingly multifarious when host heterogeneities and age-structure are included, or a third competing parasite species is considered [34,57]. As we continue to build upon our model, however, we do not anticipate revising the conclusions of our study.

## Conclusions

Data on co-infection prevalence is insufficient to infer upon between-species parasite interactions. Moreover, population-level intervention efficacy can be seriously compromised if between-parasite species interactions are not taken into account. We have shown how knowledge of parasite interactions can be used to facilitate infection control, even in the presence of drug resistance. Although this demonstration of indirect control through targeting a co-circulating synergist offers a novel tool for combating drug insensitivity, of greater immediacy is elucidating when and where public health might actually be compromised by injudicious control. In following the construction of classic epidemiological models, our framework offers an easy transition between the current paradigm and future studies that consider explicitly the pathogen species assemblage.

## Competing interests

The authors declare that they have no competing interests.

## Authors’ contributions

All authors developed the concept of the study. LY produced and analysed the mathematical model and wrote the first draft of the manuscript. All authors read and approved the final version of the manuscript.

## Supplementary Material

Additional file 1 **Yakob et al. Slaving and Release in Co-infection Control.** Allowing for bi-directionality in the inter-specific interactions of P1 and P2. Click here for file
