# Supplementary material for: Slaving and release in co-infection control
Source: Parasit Vectors. 2013 May 31;6:157. doi: 10.1186/1756-3305-6-157 (PMC3691829; doi:10.1186/1756-3305-6-157)
Supplement: Additional file 1 — Yakob et al. Slaving and Release in Co-infection Control. Allowing for bi-directionality in the inter-specific interactions of P1 and P2. [file 1756-3305-6-157-S1.doc]

## Supporting Material: Yakob et al. Slaving and Release in Co-infection Control

## Allowing for bi-directionality in the inter-specific interactions of P1 and P2

Calculating the invasion criteria for Parasite 2 into a population already endemic for Parasite 1 is facilitated by making the assumption that, at the introduction of Parasite 2 into the system, the force of infection of Parasite 1 originating from doubly contaminated environment is negligible relative to singly contaminated environment (λ_1_E_12_ << λ_1_E_1_) and the force of contamination of Parasite 1 originating from a doubly infected host is negligible relative to a singly infected host (β_1|12_I_12_ << β_1_I_1_). Following Choisy and de Roode (2010), the resulting equations describing the initial spread of Parasite 2 into the population are:[^1^](#_ENREF_1)

dI_2_/dt = λ_2_(E_2_ + E_12_)S + γ_1|12_I_12_ – [γ_2_ + α_2_λ_1_E_1_ + μ + μ_2_]I_2_

dI_12_/dt = α_1_λ_2_(E_2_ + E_12_)I_1_ + α_2_λ_1_E_1_I_2_ – (γ_1|12_ + γ_2|12_ + γ_12_ + μ + μ_12_)I_12_

dE_2_/dt = (β_2_I_2_ + β_2|12_I_12_)E + ν_1_E_12_ – (ν_2_ + β_1_I_1_)E_2_

dE_12_/dt = (β_2_I_2_ + β_2|12_I_12_)E_1_ + (β_1_I_1_)E_2_ – (ν_1_ + ν_2_)E_12_

In next generation matrix notation,[^2^](#_ENREF_2) this infection subsystem becomes:

T = [ 0 λ_2_Ŝ 0 λ_2_Ŝ

β_2_Ê 0 β_2|12_Ê 0

0 α_1_λ_2_Î_1_ 0 α_1_λ_2_Î_1_

β_2_Ê_1_ 0 β_2|12_Ê_1_ 0 ]

Σ = [-(γ_2_+α_2_λ_1_Ê_1_+μ+μ_2_) 0 γ_1|12_  0

0 -(ν_2_+β_1_Î_1_) 0 ν_1_

α_2_λ_1_Ê_1_ 0 -(γ_1|12_+ γ_2|12_ +γ_12_+μ+μ_12_) 0

0 β_1_Î_1_ 0 -(ν_1_+ν_2_) ]

The squared dominant eigenvalue of –TΣ^-1^ is:

R_2|1_ = [ λ_1_λ_2_(Ê_1_α_2_β_2|12_Ŝ + Ê_1_Î­_1_(α_2_^2)β_2|12_ + β_2_Ŝ(γ_1|12_+γ_2|12_+γ_12_+μ+μ_12_)

+ Î_1_α_2_(β_2|12_μ_2_+β_2|12_μ+β_2_γ_1|12_ + β_2|12_γ_2_) ]

/ [ ν_2_(Ê_1_α_2_λ_1_(γ_12_+γ_2|12_+μ+μ_12_) + μ_12_(γ_2_+μ+μ_2_) + μ_2_(γ_1|12_+ γ_2|12_+ γ_12_+μ)

+ μ(γ_2_+γ_1|12_+ γ_2|12_+ γ_12_+μ) + γ_2_(γ_1|12_+ γ_2|12_+ γ_12_)]

**Supporting Material Figures**

**
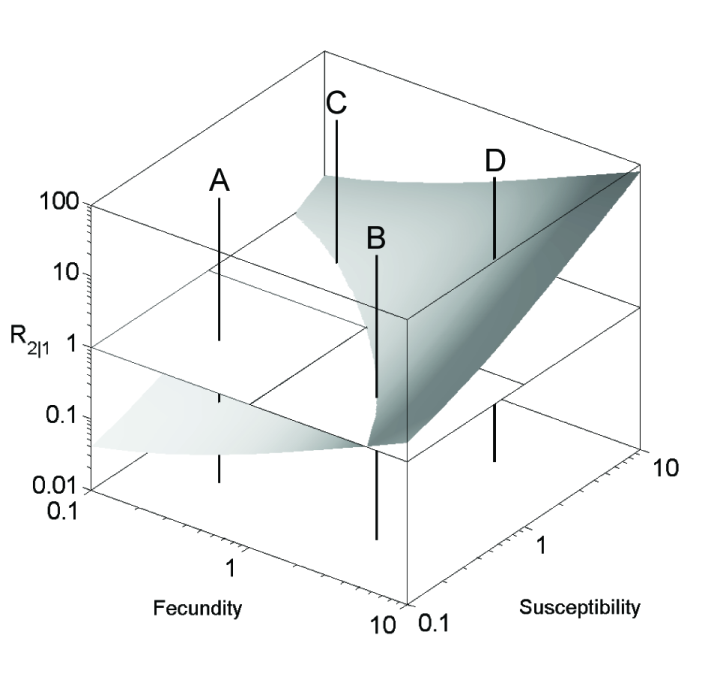
**

S Figure 1. The outbreak threshold of a gastro-intestinal nematode introduced into a host population already endemic for a heterologous nematode (*R_2|1_*). The resident nematode can either enhance or reduce host susceptibility to the invading nematode and either increase or decrease its fecundity once established. In the absence of any interspecific interaction, *R_2|1_* is set to equal 1. The four vertical lines correspond to the four qualitatively different interaction combinations explored further in this analysis. The effect of Parasite 1 on Parasite 2 is as follows: A) P1 reduces both susceptibility to, and fecundity of, P2 (-/-); B) P1 reduced susceptibility to, but increases fecundity of, P2 (-/+); C) P1 increases susceptibility to, but reduces fecundity of, P2 (+/-); D) P1 increases susceptibility to, and increases fecundity of, P2 (+/+).


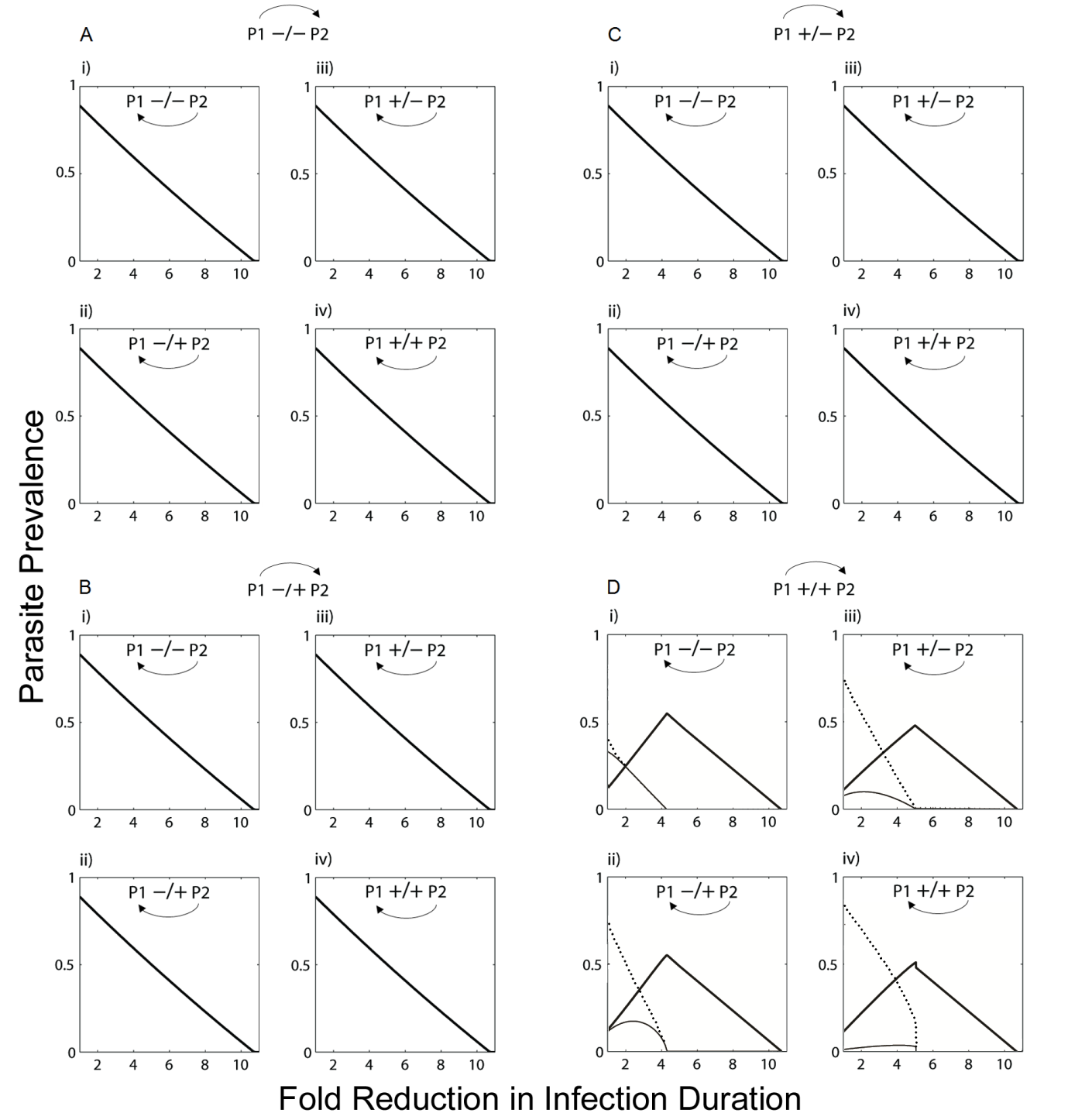


S Figure 2. Parasite prevalence (P1 is the thick line, P2 is the thin line and co-infection is the broken line), under simultaneous control of both parasites (x-axis is the fold reduction in both parasite infection durations). The effect of Parasite 1 on Parasite 2 is as follows: A) P1 reduces both susceptibility to, and fecundity of, P2 (-/-); B) P1 reduced susceptibility to, but increases fecundity of, P2 (-/+); C) P1 increases susceptibility to, but reduces fecundity of, P2 (+/-); D) P1 increases susceptibility to, and increases fecundity of, P2 (+/+). The equivalent effect of Parasite 2 on Parasite 1 is represented by the smaller subplots (respectively i, ii, iii and iv).


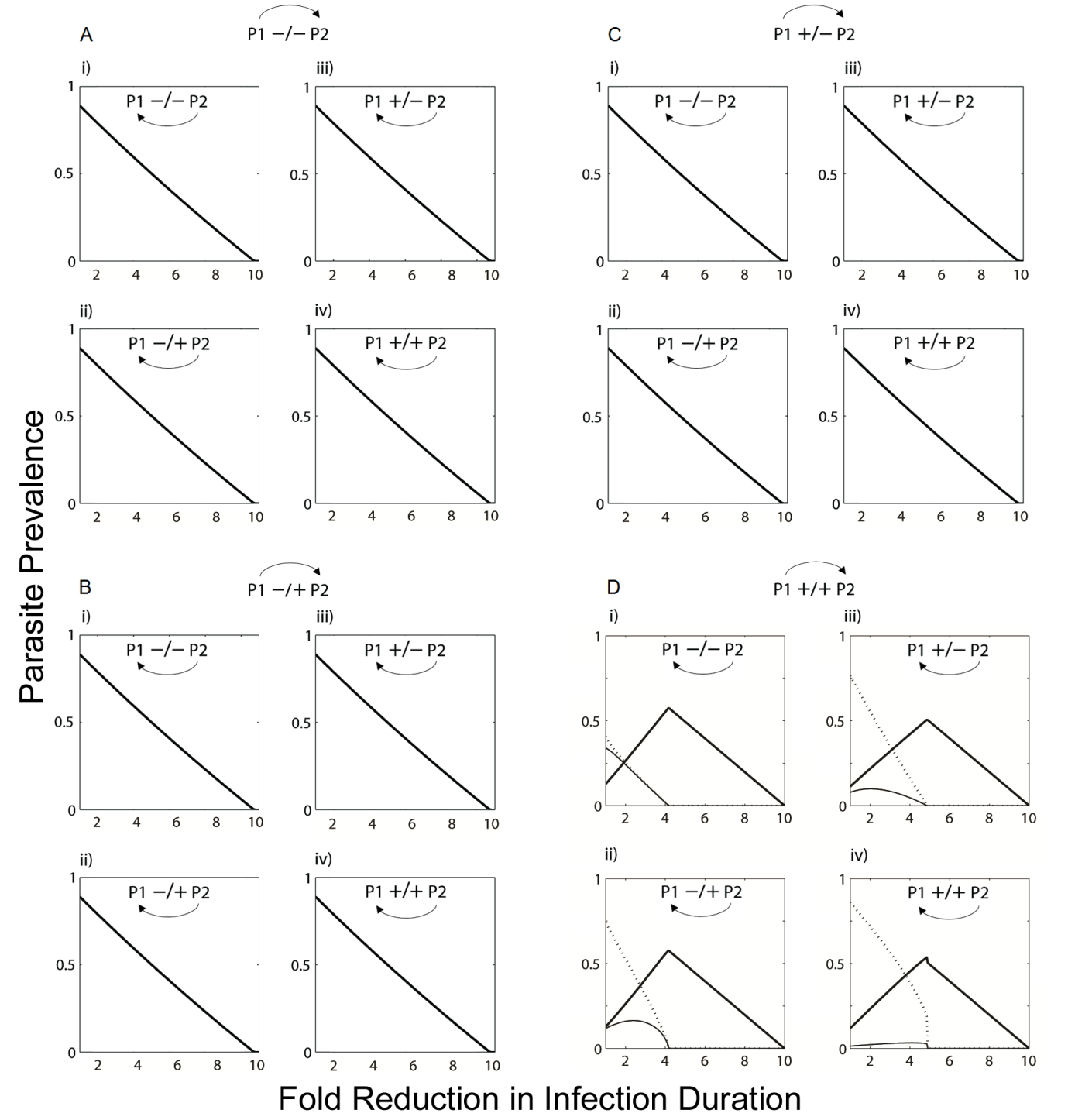


S Figure 3. Parasite prevalence (P1 is the thick line, P2 is the thin line and co-infection is the broken line), under simultaneous control of both parasites (x-axis is the fold reduction in environmental contamination with both parasites). The effect of Parasite 1 on Parasite 2 is as follows: A) P1 reduces both susceptibility to, and fecundity of, P2 (-/-); B) P1 reduced susceptibility to, but increases fecundity of, P2 (-/+); C) P1 increases susceptibility to, but reduces fecundity of, P2 (+/-); D) P1 increases susceptibility to, and increases fecundity of, P2 (+/+). The equivalent effect of Parasite 2 on Parasite 1 is represented by the smaller subplots (respectively i, ii, iii and iv). Complete elimination occurs by driving the basic reproduction number of P1 below unity, which is achieved by reducing environmental contamination with P1 by a factor of (β_1_λ_1_)/[ν_1_(γ_1_+μ+μ_1_)].

1 Choisy, M. & de Roode, J. C. Mixed Infections and the Evolution of Virulence: Effects of Resource Competition, Parasite Plasticity, and Impaired Host Immunity. *American Naturalist* **175**, E105-E118 (2010).

2 Diekmann, O., Heesterbeek, J. A. P. & Roberts, M. G. The construction of next-generation matrices for compartmental epidemic models. *Journal of The Royal Society Interface* **7**, 873-885, doi:10.1098/rsif.2009.0386 (2010).
